# Phytochemical and Biological Profile of *Moricandia arvensis* (L.) DC.: An Inhibitor of Pancreatic Lipase

**DOI:** 10.3390/molecules23112829

**Published:** 2018-10-31

**Authors:** Mariangela Marrelli, Federica Morrone, Maria Pia Argentieri, Lucia Gambacorta, Filomena Conforti, Pinarosa Avato

**Affiliations:** 1 Department of Pharmacy, Health and Nutritional Sciences, University of Calabria, I-87036 Rende, Italy; mariangela.marrelli@unical.it (M.M.); fede.morrone.90@hotmail.it (F.M.); filomena.conforti@unical.it (F.C.); 2 Department of Pharmacy-Drug Science, University of Bari “Aldo Moro”, I-70125 Bari, Italy; mariapia.argentieri@uniba.it; 3 Institute of Sciences of Food Production, National Research Council (ISPA-CNR), 70126 Bari, Italy; lucia.gambacorta@ispa.cnr.it

**Keywords:** antioxidant, flavonoids, glucosinolates, obesity, pancreatic lipase

## Abstract

Pancreatic lipase, a key enzyme for lipid absorption, is one of the most important targets for the treatment of obesity, while natural compounds have recently attracted much interest as potential inhibitors of this enzyme. Here, in an attempt to find new effective agents, the methanolic extract from *Moricandia arvensis* (L.) DC. and its sub-extracts were investigated for their potential inhibitory activity. The ability to inhibit pancreatic lipase was verified through the in vitro evaluation of the prevention of *p*-nitrophenyl caprylate hydrolysis. The antioxidant activity was also verified by means of DPPH and β-carotene bleaching tests. Compositional profiling revealed that flavonoid glycosides were the main specialized metabolites present in the methanolic extract from the aerial parts of the plant with kaempferol and quercetin representing the two *O*-glycosylated aglycones. Kaempferol-3-*O*-β-(2″-*O*-glucosyl)-rutinoside and kaempferol-3-*O*-*a-*arabinosyl-7-*O*-rhamnoside were the most abundant flavonols. The crude methanolic extract and the dichloromethane and ethyl acetate sub-extracts showed a strong lipase inhibitory activity, with IC_50_ values of 2.06 ± 0.02, 1.52 ± 0.02 and 1.31 ± 0.02 mg/mL, respectively. The best capacity to scavenge DPPH radical was detected for the ethyl acetate sub-extract (IC_50_ = 171.9 ± 1.0 µg/mL), which was also effective in protecting linoleic acid from peroxidation (IC_50_ = 35.69 ± 2.30 µg/mL). Obtained results support the hypothesis that *M. arvensis* can be a source of bioactive phytochemicals for the pharmacological inhibition of dietary lipids absorption.

## 1. Introduction

The World Health Organization (WHO) defines the overweight and obesity as abnormal or excessive fat accumulation states representing a health risk. Overweight and obesity are major factors of risk for a number of chronic diseases, including diabetes, cardiovascular diseases and cancer. There are many ways to prevent or control obesity, one of which is the inhibition of gastrointestinal lipase. Orlistat is a clinically available anti-obesity drug that acts as inhibitor of gastric and pancreatic lipase and has been shown to block the absorption of dietary fats of about 30% at the therapeutic oral dosage of 120 mg *per* three times a day. The use of this drug is being gradually decreased due to its side effects such as insomnia, constipation, vomiting, emesis, headache, and stomachache [1,2].

For this reason, there is an increasing demand for alternative inhibitors of pancreatic lipase, such as molecules of plant origin. As a consequence, more trials have been conducted with herbal medicines reported to possess anti-obesity potential in vitro and in vivo. These herbal medicines obtained interest due to their natural origin, cost effectiveness and minimal side effects [3,4,5,6].

The genus *Moricandia* DC. (Brassicaceae family), includes eight species distributed in the Mediterranean regions. Only one species is endemic to Italy: *Moricandia arvensis* (L.) DC. [7]. The leaves of *M. arvensis* are used as traditional medicine and in traditional cooking. Decoctions of leaves and stems were for example employed in the treatment of syphilis and scorbut [8].

From a phytochemical point of view in *M. arvensis* an indole derivative (3-indolylethylene oxide), glucosinolates (2-hydroxy-3-butenyl-, 3-indolylmethyl- and 1-methoxy-3-indolylmethyl- glucosinolate), antocyanins and fatty acids have been isolated and characterized [9,10,11,12].

For the first time, Braham and coworkers identified [13] in the methanolic extract from the violet flowers of the plant, new phenolic glycosides, namely, quercetin 3,4′-di-*O*-β-d-glucopyranoside-7-*O*-α-l-rhamnopyranoside, also known as moricandin, β-d-glucopyranosyl-4-*O*-β-d-glucopyranosyl-caffeate, methyl-3-*O*-β-d-glucopyranosyl-5-hydroxycinnamate and β-d-glucopyranosyl-4-*O*-β-d-glucopyranosyl-benzoate. In addition, the known compounds β-d-glucopyranosyl-4-hydroxybenzoate, methyl-4-*O*-β-d-glucopyranosyl-caffeate, 1-*O*-caffeoyl-β-d-glucopyranoside and 2-phenylethyl-β-d-glucopyranoside. Recently, some flavone (5,7-dihydroxy-3,6,4′-trimethoxyflavone; 5,7,4′-trihydroxy-3,6,8,3′-tetramethoxy-flavone; 3, 3′,4′,5,7-pentahydroxy-flavone) and flavonol (3-glucosyl-3′,4′,5,7-tetrahydroxy-flavonol and kaempferol-3-digalactopyranoside) constituents have been identified in a water extract from the leaves of *M. arvensis* collected in the Algerian Sahara [14].

In the limited biological investigations on this species, the extracts prepared from the roots and leaves of *M. arvensis* were reported to inhibit the genotoxicity induced by H_2_O_2_. In addition, a study on the antioxidant potential of root and leaf extracts under different antioxidant tests indicated that the root extract possesses a potent antioxidant activity namely through its capacity to transfer electrons [15]. An aqueous extract from *M. arvensis* also showed anti-genotoxic effect suggesting that the plant has the potential to protect DNA from the action of nitrofurantoin and free radicals generated by H_2_O_2_ [16]. *M. arvensis* subsp *eu-arvensis* collected from the southern region of Tunisia showed antimutagenic effects against sodium azide using Ames *Salmonella* tester strains TA100 and TA1535 with and without metabolic activation (S9), and while using the plasmid pBluescript DNA assay [17]. In addition, Skandrani and collaborators demonstrated that the chloroform extract from *M. arvensis* inhibits growth of B16-FO melanoma cells and human leukemic cells (K562) [18,19].

Aims of the present study were to characterize for the first time the phytochemical composition of the methanolic extractives of *M. arvensis* aerial parts collected wild in Calabria region, Italy, and determine for the first time their effect on lipid absorption trough inhibition of pancreatic lipase and antioxidant activity.

## 2. Results

### 2.1. Phytochemical Profile

Dried *M. arvensis* (L.) DC. aerial parts were extracted with methanol (MeOH) by maceration. Extraction yield was 17.8%. A portion of the obtained crude extract was then fractionated using solvents with increasing polarity: *n*-hexane (*n*-Hex), dichloromethane (DCM) and ethyl acetate (EtOAc). Percentage yields accounted for 1.1%, 0.6% and 0.6%, respectively (referred to dry plant material).

Apolar constituents present in the *n*-Hex and DCM sub-extracts were identified by means of gas chromatography–mass spectrometry (GC–MS) analyses (Table 1). Six fatty acids were detected in the *n*-Hex fraction, being palmitic, stearic and myristic acids the most abundant ones (0.9%, 0.7% and 0.6%, respectively). Some of these fatty acids have been already reported by Zeraib and coworkers, who identified them in the essential oil from the aerial parts from *M. arvensis* (L.) DC. grown in Algeria [20]. The diterpene neophytadiene (1.0%) was also found in this extract, together with the three phytosterols β-Sitosterol, 22,24-dimethylcholesterol and stigmasta-3,5-dien-7-one.

Different terpenoids were identified in the DCM fraction. Among them, the monoterpene lactone loliolide and the isoprenoid ketone hexahydrofarnesyl acetone were particularly abundant (6.1% and 3.4%, respectively).

Total phenolic and total flavonoid contents of the crude MeOH extract of *M. arvensis* were also assessed and amounted to 92.5 ± 1.0 mg/g and 18.34 ± 0.07 mg/g, respectively. The amounts were expressed as chlorogenic acid and quercetin equivalents per g of dry plant material.

The presence of phenolics in the MeOH crude extract was also indicated by the preliminary compositional inspection with NP-PEG sprayed TLC which showed some intense orange-yellow and yellow-green spots possibly due to the presence of flavonol glycosides of quercetin and kaempferol, respectively [21]. 

The phenolics profile of the MeOH extract as obtained by HPLC-PDA consisted of a major group of 7 components eluting between 13 and 20 min, which varied in their relative quantities. Combination of analytical data from HPLC-PDA and HPLC-HRMS (Table 2) indicated the presence of flavonoids; in particular, UV-spectra of eluted components showing two major absorption peaks in the range of 240–280 nm (A-ring, benzoyl system, Band I) and 330–380 nm (B-ring, cinnamoyl system, Band II) were consistent with the structure of flavonols or flavones. A closer inspection of these compounds suggested that they were flavonol derivatives of kaempferol (264, 294 *sh*, 323 *sh*, 364) and quercetin (256, 269 *sh*, 300 *sh*, 370). This was further confirmed by the fragmentation pattern observed in the mass spectra (Table 2), that is constituents were characterized by [Aglycone + H]^+^ ions at *m*/*z* 287 or 303, corresponding to kaempferol and quercetin respectively. Moreover, as already reported for other Brassicaceae [22,23,24,25,26], they were present as mono-, di- and tri-glycosides with, in some cases, sophorose (β-1,2-linked glucose) and rutinose (rhamnosyl-(α1 → 6)-glucose) as the disaccharide moieties (Table 2). Diagnostic fragments deriving from the loss of substituted sugars (−162 or 146 Da) from the protonated molecule also indicated that the identified compounds were all *O*-glycosides. Moreover, their UV spectra showed a hypsochromic shift of Band I compared with that of the aglycones revealing a substitution at 3- or 3,7-position in the molecule, that is UV maxima around 266 nm and 340–348 nm were attributed to kaempferol glycosides, while UV maxima centred around 256 nm and 350–354 nm were attributed to quercetin glycosides [23,25,26,27,28,29].

Consistently with the above UV and MS observations and by comparison with data from the literature [23,25,26,27,28,29], the following phenolics have been tentatively identified (Table 2): **1**, **2**, **5**, **6** and **7** displayed the same [Aglycone + H]^+^ ion at *m*/*z* 287 indicating that they were all derivatives of kaempferol. Compound **1** (Rt = 14.08; 1.11 ± 0.02 mg/mL) had a pseudomolecular ion at *m*/*z* 757 [M + H]^+^ which produced fragment ions deriving from the losses of −146 Da, −162 Da, −308 (162 + 146) Da and −(308 + 162) Da indicating the presence of two hexoses and a deoxyhexose moiety. Moreover, the simultaneous loss of the diglycosyl residue −308 Da revealed the presence of the disaccharide moiety rutinose. Based on the above data, **1** was identified as kaempferol-3-*O*-β-(2″-*O*-glucosyl)-rutinoside. Compound **2** (Rt = 14.60; 0.87 ± 0.01 mg/mL) showed a parent ion at *m*/*z* 727 [M + H]^+^ and fragment ions at −132 Da (the loss of a pentose), −146 Da (deoxyhexose), and a further loss of −162 (hexose): The compound was identified as kaempferol-3-*O*-β-(2″-*O*-xylosyl-6″-*O*-rhamnosyl)-glucoside. Compound **5** (Rt = 18.26; 0.28 ± 0.01 mg/mL) with a protonated pseudomolecular ion at *m*/*z* 757, was clearly identified as a triglycoside containing a sophorose unit (−324 Da, −2 × 162 Da) and one deoxyhexose moiety (−146 Da). Considering that a glycan substituent at the 3-position in a protonated flavonoid is more readily lost compared to the 7-position, compound **5** was identified as kaempferol-3-*O*-β-sophoroside-7-*O*-α-rhamnoside. Compound **6** (Rt = 18.28; 0.41 ± 0.01 mg/mL) was characterized by a pseudomolecular ion at *m*/*z* 595, an intense fragment due to the loss of a hexose (−162 Da) and a further loss of a deoxyhesose (−146 Da). It was identified as kaempferol-3-*O*-β-glucosyl-7-*O*-α-rhamnoside. Compound **7** (Rt = 18.95; 1.32 ± 0.02 mg/mL with a pseudomolecular ion at *m*/*z* 565 displayed the loss of a pentose (−132 Da) and a dehoxyhesose (−146 Da). It was identified as kaempferol-3-*O*-β-arabinosyl-7-*O*-α-rhamnoside. 

Compounds **3** (Rt = 16.47; 0.10 ± 0.01 mg/mL) and **4** (Rt = 16.81; 0.41 ± 0.31 mg/mL) shared the same [Aglycone + H]^+^ ion at *m*/*z* 303 and they were then identified as derivatives of quercetin (Table 2). Mass spectrum of **3** showed a pseudomolecular ion at *m*/*z* 773 and an abundant fragment ion at *m*/*z* 433, deriving from the loss of −324 Da (162 Da × 2) and suggesting the presence of a sophorose unit; in addition, the further loss of −146 Da indicated the presence of a deoxyhexose in the molecule. Compound **3** was then identified as quercetin-3-*O*-β-sophoroside-7-*O*-α-rhamnoside. Compound **4**, with a pseudomolecular ion at *m*/*z* 611, a dominant fragment consisting with the loss of a hexose (−162 Da) and a further ion indicating the loss of 146 Da, was instead identified as quercetin-3-*O*-β-glucosyl-7-*O*-α-rhamnoside.

Aerial parts of *M. arvensis* have also been investigated for their content of glucosinolates (GLSs). As illustrated in Table 3, from their mass spectra fragmentation seven GLSs have been identified, all of the aliphatic structural type, but one aromatic, glucotropaeolin. Among the aliphatic GLSs, two alkenyl sulfinyl GLSs (glucoviorylin and glucoibervirin) have been characterized. Quantitation of the GLSs extracted from the aerial parts of *M. arvensis* indicated that the relative amount of these metabolites was quite low, ranging from 1.38 × 10^−7^ (±6.24 × 10^−9^) μmol/g of drug (glucoiberverin) to 8.20 × 10^−8^ (±3.40 × 10^−9^) μmol/g of drug (3-hydroxypropyl-GLS). Glucoviorylin, accounting for 5.87 × 10^−7^ (±1.28 × 10^−8^) μmol/g of drug, was another dominant component, followed by 3-hydroxybutyl-GLS and gluconapin amounting to 3.12 × 10^−7^ (±1.54 × 10^−8^) and 3.08 × 10^−7^ (±1.79 × 10^−8^) μmol/g of drug respectively.

### 2.2. Antioxidant Activity

The antioxidant potential of *M. arvensis* (L.) DC. MeOH extract and its sub-extracts was assessed by means of in vitro DPPH and β-carotene bleaching tests. A modest capacity to scavenge DPPH radical was detected for the crude MeOH extract, with an IC_50_ value equal to 355.5 ± 7.9 μg/mL (Table 4).

The EtOAc sub-fraction was the most active, with an IC_50_ value of 171.9 ± 1.0 μg/mL. On the contrary, the *n*-Hex sub-extract was not effective, and a very low radical scavenging activity was observed for the DCM sample (IC_50_ = 870.7 ± 15.9 μg/mL).

The crude MeOH extract and its EtOAc sub-fraction were instead very effective in protecting linoleic acid from peroxidation, as assessed by the β-carotene bleaching test. Both samples demonstrated a good inhibitory activity, with IC_50_ values equal to 37.36 ± 3.06 and 35.69 ± 2.30 μg/mL after 30 min of incubation. There were no differences among these two samples, as confirmed by post-hoc comparison (Bonferroni test). The EtOAc sample was still effective after 60 min of incubation (IC_50_ = 63.92 ± 2.51 μg/mL), even if the biological potential was significantly decreased.

The antioxidant activity of the leaves of *M. arvensis* collected in Tunisia has been already reported by Skandrani and coworkers [30], who tested different extracts obtained with soxhlet extraction. In particular, the methanolic extract showed a strong radical scavenging activity (IC_50_ = 2.25 µg/mL). On the contrary, the aqueous extract was not an effective DPPH radical scavenger, inducing 32.65% of DPPH inhibition at a concentration of 100 µg/mL.

### 2.3. Pancreatic Lipase Inhibition

Herbal remedies have gained much attention as natural effective drugs against obesity. A potential anti-obesity activity has been already demonstrated for different classes of phytochemicals such as polyphenols, phytosterols, saponins and terpenes [31,32]. Specialized metabolites are able to play a role in bodyweight control by different mechanisms of action. Inhibition of lipid absorption is one of the most important strategy in the treatment of obesity [33].

In the present paper, the MeOH extract from the aerial parts of *M. arvensis* and its sub-extracts (*n*-Hex, DCM and EtOAc) were tested for their ability to inhibit pancreatic lipase, a key enzyme for dietary fat absorption. Porcine pancreatic lipase activity was measured using *p*-nitrophenyl caprylate (NPC) as a substrate.

**Figure 1:**
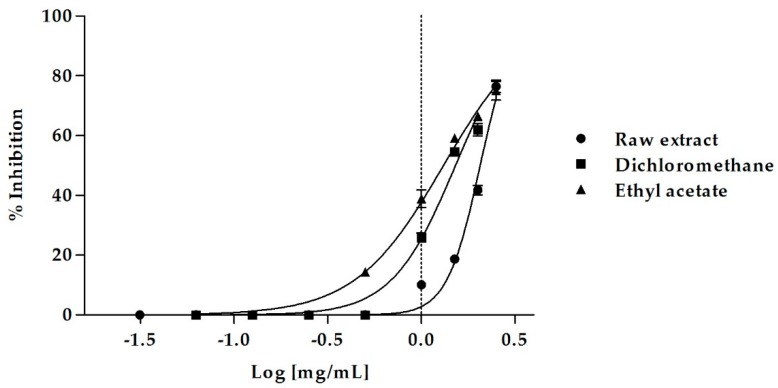
Dose-dependent pancreatic lipase inhibition induced by *M. arvensis* (L.) DC. MeOH extract and its sub-extracts.

An excellent biological activity was observed both for the crude MeOH extract and its sub-extracts, with the only exception being the *n*-Hex sub-fraction, that was not effective (Figure 1).

The raw MeOH extract caused a dose-dependent inhibition of lipase activity, inducing 76.46 ± 2.05% of inhibition at the concentration of 2.5 mg/mL and 41.77 ± 1.63% of inhibition at 2 mg/mL. At this last concentration, the EtOAc sub-extract was even more active, causing 66.44 ± 0.75% of enzyme inhibition, which decreased to 59.24 ± 0.07% and 38.91 ± 2.84% at the concentrations of 1.5 mg/mL and 1 mg/mL, respectively. Also, the DCM sub-extract showed an excellent biological activity, inducing 61.98 ± 2.03% of inhibition at a concentration of 2 mg/mL.

The MeOH extract of *M. arvensis* aerial parts was able to effectively inhibit pancreatic lipase with an IC_50_ value of 2.06 ± 0.02 mg/mL (Table 5). The DCM and the EtOAc sub-fractions were even more active, with IC_50_ values of 1.52 ± 0.02 and 1.31 ± 0.02 mg/mL, respectively. The EtOAc sub-extract was the most effective, with an excellent inhibitory activity on pancreatic lipase (Bonferroni post-hoc test). The *n*-Hex sub-extract was the only fraction that showed no activity.

## 3. Discussion

The Brassicaceae botanical family (syn. Cruciferae) are a rich source of valuable phytochemicals displaying health promoting properties [22]. In particular, vegetables belonging to this family are characterized by the presence of sulfur compounds responsible for the sharp flavor produced when tissues of the plant are powdered or crushed [10,34]. They are the glucosinolates, β-thioglucoside-*N*-hydroxysulfates deriving from the amino acid metabolism. In addition, Brassicaceae contain phenolics, including typical structural types of flavonoids [23], such as glycosylated flavonols of quercetin, kaempferol and isorhamnetin. 

*M. arvensis* which belongs to the botanical family of Brassicaceae, it was therefore expected to contain glucosinolates and phenolic compounds. Previous investigations on the content of these specialized metabolites in the seeds [34] as well as in mature tissues [14] of *M. arvensis* from Algeria indicated the presence of GLSs of the indole structural type. Adversely, the analysis of the aerial parts of our plants collected at anthesis displayed a different composition of GLSs, only including aliphatic GLSs and one benzyl derivative (Table 3). It has been reported in several studies [22,34] that chemical types and amounts of GLSs is highly variable depending on the plant species, the plant organ, its developmental stage and environmental influences, therefore GLSs compositional profile found by us is possibly due to a natural variation under the above factors.

Methanol extract of *M. arvensis* used in the bioassays contained flavonoids which shared the general chemical features found for these phytochemicals in several other Brassicaceae [22,23,24,25,26,27,28,29,35], that is the occurrence of quercetin and kaempferol as the main aglycones, the *O*-glycosylation and the presence of sophorose and rutinose as common disaccharides. Compositional differences were however observed compared to previous reports on *M. arvensis* [12,13,14], which should not be considered unexpected, taking into account the type of plant tissue and the extraction procedure/solvent used in this investigation.

Flavonoids display interesting biological properties such as anti-inflammatory, vascular protection, enzyme inhibition, some of them are cytotoxic and antitumoral; they can be antiallergic or antispasmodic which explains their use in therapy.

Contribution of these phytochemicals to health improvement has been generally related to their antioxidant capacity associated with their, more or less, hydroxylated structure and glycosylation pattern [23,36]. In particular the two aglycones quercetin and kaempferol have received considerable attention for their strong antioxidant potential due to the di-hydroxylated B ring and the presence of the 2–3 double bond in conjugation with the 4-carbonyl group in the C ring. Consistently, the MeOH extract of *M. arvensis* rich in derivatives of quercetin and kaempferol showed a fairly good radical scavenging capacity through the DPPH and β-carotene bleaching test (Table 4).

Among other biological activities, these molecular properties make quercetin and kaempferol good inhibitors of lipid peroxidation and it has been reported that quercetin can influence adipogenesis and apoptosis through a molecular mechanism that involves regulation of the hepatic gene expression related to lipid metabolism. A positive influence of dietary quercetin on hyperglycemia and dyslipidemia has also been demonstrated in animal models with type 2 diabetes mellitus [37,38]. In addition, a protective effect against diabetes has been demonstrated for kaempferol in different animal and in vitro experimental models [39]. Glycosylation of flavonoid aglycones has been also shown to contribute to the anti-obesity function of these phytochemicals [36]. In particular, a strong inhibition against lipid accumulation in liver cells has been proved in vitro for some glycosides of kaempferol [36,39].

An important target to fight obesity includes the development of inhibitors of pancreatic lipase, a key enzyme in the digestion and absorption of dietary fats. Inhibition of this enzyme can affect the lipolysis rate and reduce fat absorption. Several studies have shown that natural products and plant extracts can be good inhibitors of pancreatic lipase [3,5,6,31,32,33]; among them, polyphenols or plant extracts rich in polyphenols represent the major class of phytochemicals identified for the inhibition of pancreatic lipase [40]. Thus, some flavonoids (quercetin, quercetin arabinoside, quercitrin, rutin, etc.) have been described to possess inhibitory effect against pancreatic lipase. Moreover, a previous investigation by us [41] on the efficacy as antilipidemic of MeOH extracts from *Capparis sicula* subsp. *sicula* and *C*. *orientalis* showed that they were rich in rutin (quercetin-3-*O*-rutinoside) and had a pronounced dose-dependent inhibitory activity toward pancreatic lipase.

In agreement with the above data, this study provides evidence that *M. arvensis* rich in glycosylated flavonols of quercetin and kaempferol had a very interesting lipase inhibitory effect and can represent another valuable source for developing novel pharmaceutical anti-obesity products. According to the promising in vitro effectiveness, *M. arvensis* is a good candidate for further studies. Obtained results motivate a deeper investigation of this species in order to identify the single active principles responsible for the biological properties observed in in vitro experiments and possibly showing, a much higher lipase inhibitory activity. 

Further in vivo studies are needed to verify the effectiveness of this plant extract and its sub-fractions and main constituents in inhibiting dietary lipid absorption and they will be of help to validate the potential health benefits of these samples in the treatment of obesity. 

## 4. Materials and Methods

### 4.1. Collection of Plant Material and Preparation of the Extracts

Wild *M. arvensis* aerial parts were collected in Calabria, Italy, in the Reggio Calabria district (RC), in June 2015 (leg. F. Conforti, det. F. Conforti) and dried under the shade for 15 days. A voucher specimen is deposited in the Herbarium of the University of Calabria (CLU 22692). Dried plant material (74 g) was extracted with methanol (2 L) through maceration (48 h × 3 times) at room temperature. The resulting total MeOH extract was then filtered, dried under reduced pressure and weighed to determine the extraction yield. A fraction of the extract was solubilized in a mixture of methanol:water (9:1) and partitioned between *n*-hexane (*n*-Hex), dichloromethane (DCM) and ethyl acetate (EtOAc).

### 4.2. GC–MS Analysis

*G*as chromatography–mass spectrometry (GC–MS) analyses allowed the identification of chemical compounds present in the *n*-Hex and DCM sub-extracts of *M. arvensis*. The apparatus consisted of a Hewlett-Packard 6890 gas chromatograph equipped with an SE-30 and a selective mass detector (model 5973, Hewlett Packard). Analyses were run according to the same operating conditions previously described [42]. A capillary column 100% dimethylpolysiloxane, 30 m length, 0.25 mm in diameter and 0.25 μm film thickness was used. Analyses were performed using a programmed temperature from 60 to 280 °C and compounds were identified by comparison of their mass spectra with those present in the Wiley 138 library data of the GC–MS system.

### 4.3. Determination of Total Phenolic Content (TPC) and Total Flavonoid Content (TFC)

Total polyphenolic content was evaluated by a variation of the Singleton and Rossi’s method, as previously described [43] using the Folin–Ciocalteu (FC) reagent, consisting of a mixture of phosphomolybdic and phosphotungstic acids. *M. arvensis* MeOH extract was mixed with distilled water, sodium carbonate solution and Folin-Ciocalteau reagent. After 2 h the absorbance of the blue color produced was measured at 765 nm. Chlorogenic acid was used as standard and total phenolic content was expressed in mg *per* g of dry plant material.

Total flavonoid content was instead determined using a method based on the formation of a flavonoid-aluminum complex [44].

### 4.4. Glucosinolates Extraction and Desulfation

A finely pulverized sample (400 mg) of dried aerial parts of *M. arvensis* was extracted (ISO-Method 9167-1, 1992) with boiling MeOH:H_2_O (70:30%) to isolate glucosinolates (GLSs). Extraction was carried out in duplicate. As previously described [45], the crude extract (2.5 mL) was then applied to a SPE column (0.8 × 4 cm; BIO-RAD Polyprep) packed with DEAE Sephadex A-25 anion-exchange resin previously equilibrated with 0.5 M Na-acetate buffer at pH 5.0 (1.6 g of resin and 25 mL of buffer) and pre-washed with H_2_O. A preliminary clean up of the extract to eliminate co-extraction products was performed by flushing the cartridge with MeOH 70% (2 × 2 mL) followed by H_2_O (2 × 2 mL).

Desulfation was carried out by overnight incubation of GLSs retained by the DEAE resin with a water solution of *H. pomatia* type-1 sulfatase (0.0025 g of enzyme in 6 mL of H_2_O). Desulfated glucosinolates (DGLSs) were then recovered from the column by H_2_O elution (2 × 2 mL) and stored for further analyses. Desulfation procedure was run in duplicate. All the steps of GLSs purification and derivatization were run with a SPE Extraction Manifold-20 position from Waters (Milford, MA, USA).

GLSs elution and desulfation was monitored by TLC (iPrOH:AcOEt:H_2_O, 7:1:2, *v*/*v*); products were first visualized under UV light at 254 and 366 nm and then revealed by spraying TLC plates with phosphomolibdyc acid reagent (10% EtOH).

### 4.5. Chemical Analysis and Quantitation of Glucosinolates

Glucosinolates as DGLSs were analyzed by HPLC according to [46]. A Waters 600 HPLC system equipped with a Photodiode-Array-Detector, PDA 2998, was used to separate the compounds. Data were processed with Empower^TM^ 2 Waters Software. A Phenomenex Gemini–C18 reversed phase column (250 × 4.6 mm, 5 µm particle size), equipped with a Security Guard-C18 cartridge (4 mm × 3 cm, 5 μm particles) was employed for the separation, using the following elution system: solvent A, H_2_O; solvent B, MeOH. The elution gradient consisted in: 1.5% B in A, up to 60% of B at 50 min and 100% of B at 54 min; flow rate was set at 1 mL/min. For each component eluted an UV spectrum was recorded at 229 nm. Available authentic DGLSs were run in the same analytical conditions and RT and UV spectra compared for identification.

Quantitation of glucosinolates was made by the external standard method. A calibration curve was created with a set of known concentrations (0.001–0.5 mg/mL) of desulphosinigrin and desulphogluconapin in MeOH. Triplicate measurements were taken for each level in the standard curve. Linearity was determined by plotting the peak area ratio (*y*) recorded for each GLSs *vs* its concentration (*x*). The calibration curve was fitted to a linear function with the calibration coefficient *r*^2^ = 0.999 (*y* = 1*E* + 0.7*x* + 13,251) for desulphosinigrin and *r*^2^ = 0.999 (*y* = 1*E* + 0.7*x* + 13,454) for desulphogluconapin. These indicated a good linearity between peak areas and concentrations within the tested concentration range. Precision of the HPLC method was determined by repeatability (intra-day) and intermediate precision (inter-day). The intra-day precision was calculated as the relative standard deviation (RSD %) of values of retention times and peak areas from three injections of desulphosinigrin and desulphogluconapin as reference analytes in the same day, and the inter-day precision was obtained by comparing the results on three consecutive validation days. Calculated RSDs values for intra-day retention times were from 0.53 to 0.84% (desulphosinigrin) and from 0.82 to 0.22% (desulphogluconapin), while for peak areas were from 0.47 to 1.28% (desulphosinigrin) and from 1.24 to 1.04% (desulphogluconapin); calculated RSDs values for inter-day retention times were 0.20% (desulphosinigrin) and 0.90% (desulphogluconapin), while for peak areas were 0.14% (desulphosinigrin) and 0.27% (desulphogluconapin). Limit of detection (LoD) and limit of quantification (LoQ) were evaluated on the basis of the signal-to-noise ratios (S:N) of 3:1 and on 10:1 respectively. The LoD was 0.20 μg/mL and 0.16 μg/mL for desulphosinigrin and desulphogluconapin the LoQ was 0.47 μg/mL and 0.25μg/mL for desulphosinigrin and desulphogluconapin. All HPLC analyses for identification and quantitation of GLSs were run in triplicate.

### 4.6. ESI-MS/MS Analyses of Glucosinolates

Analyses were carried out using a 1100 Series Agilent LC/MSD Trap System VL equipped with an Agilent Chem-station (LC/MSD Trap-Software 4.1) for acquisition and processing of data. An ESI ion source type, both in negative and positive mode, was used with the following settings: capillary voltage, 400 V; nebulizer gas (N_2_), 15 psi; drying gas (N_2_), heated at 350 °C and introduced at a flow rate of 5 L/min. Full scan spectra were acquired over the range of 100–2200 with a scan time of 13.000 *m*/*z*/*s*. Mass/MS fragmentation was performed by isolation of the pseudomolecular ions as described in [45]. Identification was based on comparison with reference mass spectra from standard GLSs and/or by spiking with available authentic samples. 

### 4.7. HPLC of Phenolics

The MeOH extract of *M. arvensis* prepared as described in 4.1 was subjected to HPLC analysis to determine the content of phenolics.

A preliminary screening of the MeOH extract composition was carried out by TLC (precoated silica gel 60F254 aluminium plates, Merck) eluted with EtOAc:HCOOH:CH_3_CO_2_H:H_2_O (100:11:11:27, *v*:*v*). Components were visualized with phosphomoliybdic acid reagent (10% EtOH) or alternatively with Natural Products-Polyethyleneglycol reagent (NP-PEG, Sigma, St. Louis, LO, USA). TLC plates were dried off at 110 °C and then observed under visible-254 nm or in UV-366 nm light.

HPLC analyses were performed with the same apparatus described above, fitted with the same Gemini-C18 HPLC column. Analytical conditions were as follows: solvent A, H_2_O-HCOOH 0.1%, pH 2.7; solvent B, CH_3_CN-HCOOH 0.1%; elution gradient, 10–60% B in 60 min; flow rate, mL/min UV spectra of components of the extract were conventionally recorded at 210, 270, 310 and 350 nm. All analyses were run in triplicate. HPLC identification was based by spiking with available phenolics and comparison of their RT and UV spectra; chromatographic and spectroscopic data from literature were also used [26,27,28,29,35]. 

Quantitation of phenolics was made by the external standard method. A 7-level calibration (0.0078–0.5 mg/mL MeOH) curve was created by injection of isoquercitrin (quercetin-3-*O*-β-glucoside) and kaempferol-3-*O*-β-rutinoside. Triplicate measurements were taken for each point in the standard curve. Linearity was determined by plotting the peak area average values recorded for each phenolic compound vs. the concentration. Correlation coefficient (*r*^2^) of the standard curve in the linear plot was *r*^2^ = 0.999 (*y* = 5*E* + 0.7*x* + 166,437) for isoquercitrin and *r*^2^ = 0.999 (*y* = 3*E* + 0.7*x* + 27,270) for kaempferol-3-*O*-β-rutinoside indicating a good linearity between peak areas and concentrations within the tested concentration range.

As for GLSs, precision of the adopted HPLC method for phenolics was determined by calculation of the intra-day and inter-day variations. Calculated RSDs values for intra-day retention times were from 0.81 to 1.04% (isoquercitrin) and from 0.18 to 1.29% (kaempferol-3-*O*-β-rutinoside), while for peak areas were from 3.83 to 1.86% (isoquercitrin) and from 0.18 to 1.29% (kaempferol-3-*O*-β-rutinoside); calculated RSDs values for inter-day retention times were 0.39% (isoquercitrin) and 0.23% (kaempferol-3-*O*-β-rutinoside), while for peak areas were 4.93% (isoquercitrin) and 1.29% (kaempferol-3-*O*-β-rutinoside). Limit of detection (LoD) and limit of quantification (LoQ) were also evaluated: LoD was 0.03 μg/mL and 0.19 μg/mL for isoquercitrin and kaempferol-3-*O*-β-rutinoside, respectively; LoQ was 0.12 μg/mL and 0.59 μg/mL for isoquercitrin and kaempferol-3-*O*-β-rutinoside, respectively. 

All HPLC analyses for identification and quantitation of phenolics were run in triplicate.

### 4.8. HPLC-HRMS Analyses of Phenolics

Analyses were performed with a benchtop single-stage mass spectrometer (Exactive) equipped with a heated electrospray ion source HESI II and coupled to an Accela HPLC system (Thermo Fisher Scientific, Waltham, MA, USA). The same Gemini C18 column and the same analytical conditions as described in 4.5 were used for these analyses. Scan range was set at 50.2 to 1003.2 *m*/*z*, with a resolution power of 10,000 FWHM. Other settings were as follows: sheath gas flow rate, 15 and 30 arbitrary units; auxiliary gas flow rate, 15 and 10 arbitrary units; capillary temperature, 300 °C; capillary voltage, 4 kV (positive ion mode) and 4.5 kV (negative ion mode). Data acquisition and processing were carried out with Xcalibur software (v2.1.0; Thermo Fisher). Structural characterization was based on comparison with obtained mass spectrometric data with those of available reference phenolics and/or mass spectra from library files [26,27,28,29,35].

### 4.9. Free Radical Scavenging Activity (FRSA) Assay

To assess the radical scavenging activity by the DPPH method, a 1 × 10^−4^ M methanolic solution of 2,2-diphenyl-1-picrylhidrazyl (DPPH) was prepared [47]. Then, 0.8 mL of this solution were mixed with 0.2 mL of each extract at different concentrations (5–1000 µg/mL) and the obtained solution were incubated at room temperature in the dark for 30 min. Absorbance was measured at 517 nm using a Perkin Elmer Lambda 40 UV/VIS spectrophotometer (Akron, OH, USA). Experiments were run in triplicate and ascorbic acid was used as positive control. Curves of percentages of inhibition vs. concentration were plotted to obtain IC_50_ concentration of the sample needed to get 50% of radical-scavenging activity.

### 4.10. β-Carotene Bleaching/Linoleic Co-Oxidation Assay

The antioxidant activity of *M. arvensis* methanolic extract and its sub-fractions was evaluated using the β-carotene-linoleic acid test system [48]. The absorbance was measured at 470 nm using a Perkin Elmer Lambda 40 UV/VIS spectrophotometer at time zero, 30 and 60 min. The antioxidant activity (AA) was calculated in terms of percent inhibition relative to the control, using the following equation:

AA (inhibition%) = [1 − (A_0_ − A_t_)/(A°_0_ − A°_t_)] × 100

where A_0_ and A°_0_ are the absorbance values measured at the initial incubation time for samples/standard and control, respectively, while A_t_ and A°_t_ are the absorbance values of the samples/standard and control, at t = 30 and 60 min, respectively.

### 4.11. In Vitro Pancreatic Lipase Assay

Pancreatic lipase inhibition activity of *M. arvensis* MeOH extract and its sub-fractions was determined according to Marrelli et al. [42]. Water solution of type II crude porcine pancreatic lipase (1 mg/mL) and a substrate solution of 4-nitrophenyl octanoate (5 mM) were prepared. The reaction was initiated by mixing 100 μL of enzyme solution, 100 μL of 4-nitrophenyl octanoate solution, 4 mL of Tris-HCl buffer (pH = 8.5) and 100 μL of the sample. This mixture was incubated at 37 °C for 25 min and then absorbance was measured at 412 nm.

### 4.12. Statistical Analysis

Experiments were carried out in triplicate. Data are presented as mean ± SEM. Biological data were fitted through nonlinear regression in order to calculate the IC_50_ values (concentration causing 50% inhibition). Graphs were built using GraphPad Prism Software (San Diego, CA, USA).

Data were first checked for normality (D’Agostino-Pearson test) and then subjected to one-way analysis of variance (ANOVA) with Bonferroni post-hoc test using SigmaStat Software (Jantel scientific software, San Rafael, CA, USA). Differences were considered significant at *p* ≤ 0.05.

## Figures and Tables

**Table 1:** Phytochemical profile of *n*-Hex and DCM sub-extracts of *M. arvensis* (L). DC. MeOH extract.

Compound ^1^	RT ^2^	RAP ^3^
***n*-Hex**		
**Fatty acids**		
Capric acid	13.073	0.3
Lauric acid	15.027	tr ^4^
Palmitoleic acid	15.119	tr
Myristic acid	16.799	0.6
Stearic acid	19.834	0.7
Palmitic acid	20.199	0.9
**Terpenes**		
Neophytadiene	17.445	1.0
**Phytosterols**		
β-Sitosterol	33.796	tr
22,24-Dimethylcholesterol	33.893	tr
Stigmasta-3,5-dien-7-one	36.054	2.1
**DCM**		
Vanillin	13.570	tr
2,4-Di-tert-butylphenol	14.627	3.4
Dihydroactinolide	14.970	1.8
Loliolide	17.107	6.1
Citronellyl acetone	17.496	tr
Hexahydrofarnesyl acetone	17.525	3.4

^1^ Listed in order of elution from SE-30 column. ^2^ RT: Retention time (as minutes). ^3^ RAP: Relative Area Percentage (peak area relative to total peak area %). ^4^ tr: Traces percentages ≤0.1%.

**Table 2:** Analytical data of phenolic compounds identified in the crude MeOH extract of *M. arvensis* aerial parts.

Number	Rt (min)	Name	UV (λmax, nm)	[M + H]^+^, *m/z* (%)	
**1**	14.08	Kaempferol-3-*O*-β-(2″-*O*-glucosyl)-rutinoside	266.6, 319.9 *sh*, 338.9	757 (100)	611 (1.0) [(M + H)-146]^+^; 595 (46) [(M + H)-162]^+^; 449 (4) [(M + H)-308]^+^; 287 (1) [(M + H)-308-162]^+^, [Aglycone + H]^+^
**2**	14.60	Kaempferol-3-*O*-β-(2″-*O*-xylosyl-6″-*O*-rhamnosyl)-glucoside	266.6, 319.9 *sh*, 338.9	727 (100)	595 (37) [(M + H)-132]^+^; 581 (2) [(M + H)-146]^+^; 433 (6) [(M + H)-162-132]^+^; 287 (2) [(M + H)-132-162-146]^+^, [Aglycone + H]^+^
**3**	16.47	Quercetin-3-*O*-β-sophoroside-7-*O*-α-rhamnoside	257.2, 268.0 *sh*, 352.0	773 (100)	611 (2) [(M + H)-162-]^+^; 449 (64) [(M + H)-162-162]^+^; 303 (18) [(M + H)-162-162-146]^+^, [Aglycone + H]^+^
**4**	16.81	Quercetin-3-*O*-β-glucosyl-7-*O*-α-rhamnoside	256.0, 268.0 *sh*, 354.4	611 (100)	449 (71) [(M + H)-162]^+^; 303 (19) [(M + H)-162-146]^+^, [Aglycone + H]^+^
**5**	18.26	Kaempferol-3-*O*-β-sophoroside-7-*O*-α-rhamnoside	266.6, 319.0 *sh*, 343.6	757 (100)	595 (2) [(M + H)-162]^+^; 433.1 (81) [(M + H)-162-162]^+^; 287.0 (28) [(M + H)-162-162-146]^+^, [Aglycone + H]^+^
**6**	18.28	Kaempferol-3-*O*-β-glucosyl-7-*O*-α-rhamnoside	266.6, 320.0 *sh*, 342.4	595 (100)	433 (78) [(M + H)-162]^+^; 287 (23) [(M + H)-162-146]^+^, [Aglycone + H]^+^
**7**	18.95	Kaempferol-3-*O*-α-arabinosyl-7-*O*-α-rhamnoside	266.6, 344.8 *sh*, 347.2	565 (100)	433 (100) [(M + H)-132]^+^; 419 (4) [(M + H)-146]^+^; 287 (23) [(M + H)-132-146]^+^, [Aglycone + H]^+^

**Table 3:** Mass data of glucosinolates identified in the crude MeOH extract of *M. arvensis* *.

Compound	[M + Na]^+^ *m*/*z*	MS/MS (%)
3-Hydroxypropyl-GLS	320	219 (2) [(M + Na)-RCNOH]^+^; 100 (100) [CH_2_CHCH_2_NCS + H]^+^; 72 (10) [CH_2_NCS]^+^
3-Hydroxybutyl-GLS	334	219 (35) [(M + Na)-RCNOH]^+^; 100 (100) [CH_2_CHCH_2_NCS + H]^+^
Gluconapin (3-Butenyl-GLS)	316	185 (3) [(M + Na)-RCNOH-H_2_S]^+^; 154 (2) [(M + Na)-Glu]^+^; 72 (10) [CH_2_NCS]^+^; 58 (19) [NCS]^+^
Isobutyl-GLS	318	186 (2) [(M + Na)-RCNOH-H_2_S]^+^; 100 (100) [CH_2_CHCH_2_NCS + H]^+^; 72 (10) [CH_2_NCS]^+^
Glucoviorylin (2-(Methylthio)-ethyl-GLS)	336	220 (38) [(M + Na)-RCNOH]^+^; 174 (2) [(M + Na)-Glu]^+^; 100 (100) [CH_2_CHCH_2_NCS + H]^+^; 72 (10) [CH_2_NCS]^+^
Glucoiberverin (3-(Methylthio)-propyl-GLS)	350	219 (15) [(M + Na)-RCNOH]^+^; 100 (100) [CH_2_CHCH_2_NCS + H]^+^; 58 (13) [NCS]^+^
Glucotropaeolin (Benzyl-GLS)	352	220 (2) [(M + Na)-RCNOH]^+^; 100 (100) [CH_2_CHCH_2_NCS + H]^+^; 72 (10) [CH_2_NCS]^+^

* Glucosinolates have been analyzed as desulphoglucosinolates.

**Table 4:** Antioxidant activity of *M. arvensis* (L.) DC. MeOH extract and its sub-extracts.

Sample	IC_50_ (μg/mL)		
	DPPH Test	β-Carotene Bleaching Test	
		30 min	60 min
MeOH extract	355.5 ± 7.9 ^c^	37.36 ± 3.06 ^b^	>100
*n*-Hex	>1000	>100	>100
DCM	870.7 ± 15.9 ^d^	>100	>100
EtOAc	171.9 ± 1.0 ^b^	35.69 ± 2.30 ^b^	63.92 ± 2.51 ^c^
Ascorbic acid *	2.00 ± 0.01 ^a^	-	-
Propyl gallate *	-	1.00 ± 0.02 ^a^	1.00 ± 0.02 ^a^

Data are expressed as mean ± SEM (*n* = 3). Different letters along column (DPPH) or between columns (β-carotene bleaching test) indicate statistically significant differences at *p* < 0.05 (Bonferroni post-hoc test). * Positive controls.

**Table 5:** Inhibition of pancreatic lipase induced by *M. arvensis* (L.) DC. MeOH extract and its sub-extracts.

Sample	IC_50_ (mg/mL)
MeOH extract	2.06 ± 0.02 ^d^
*n*-Hex	>10
DCM	1.52 ± 0.02 ^c^
EtOAc	1.31 ± 0.02 ^b^
Orlistat *	0.018 ± 0.001 ^a^

Data are expressed as mean ± SEM (*n* = 3). Different letters indicate statistically significant differences at *p* < 0.05 (Bonferroni post-hoc test). * Positive control.
